# Effects of Cyclic Adenosine Monophosphate Nanoliposomes on Growth Performance, Gut Development and Microbiota of Broilers

**DOI:** 10.3390/ani15131852

**Published:** 2025-06-23

**Authors:** Li Chen, Li Wang, Sheng Huang, Guoqi Su, Shan Jiang, Feiyun Yang, Jingxiu Huang

**Affiliations:** 1 Animal Nutrition Institute, Chongqing Academy of Animal Sciences, Chongqing 402460, China; 2 National Center of Technology Innovation for Pigs, Chongqing 402460, China

**Keywords:** cyclic adenosine monophosphate, liposomes, microbiota, the feed-to-gain ratio

## Abstract

This study aimed to investigate the effects of Nano-cAMP on growth performance, gut development, and microbiota composition in broilers. A total of 108 21-day-old yellow-feathered female chicks were randomly divided into three groups with six replicates per group and six chicks per replicate according to the principle of consistent body weight. Experimental treatments included the following: (1) CON group (basal diet), (2) cAMP group (basal diet + 0.02 g/kg cAMP), and (3) Nano-cAMP group (basal diet + 0.37 g/kg Nano-cAMP liposomes). After a 21-day experimental period, results revealed the following: Compared with the CON group, the Nano-cAMP group exhibited a significantly reduced feed-to-gain ratio (*p* < 0.05). The cAMP group exhibited a significant increase in duodenal index (*p* < 0.05), whereas the Nano-cAMP group demonstrated greater jejunal villus height (*p* < 0.05). Both treatment groups showed significant upregulation of cholecystokinin (CCK) and secretin gene expression (*p* < 0.05). Analysis of alpha-diversity indices (Chao1, Shannon, Simpson) revealed no significant differences in jejunal and cecal microbiota composition between experimental groups (*p* > 0.05). Notably, the relative abundance of *Firmicutes* significantly increased (*p* < 0.05) in the cAMP and Nano-cAMP groups, whereas *Proteobacteria*, *Gemmatimonadota*, and *Chloroflexi* significantly decreased (*p*< 0.05). The combined relative abundance of three *Lactobacillus* genera and *Bifidobacterium* was obviously elevated. Linear discriminant analysis identified *Bifidobacterium*, *Ruminococcus torques group*, and *uncultured_Thermoanaerobacterales_bacterium* as dominant genera in the intestinal tract of Nano-cAMP group. In conclusion, dietary addition of Nano-cAMP promotes jejunal development, modulates appetite hormones mRNA expression, enhances absorption capacity, increases the relative abundance of intestinal probiotics such as *Bifidobacterium* and cellulose-degrading bacteria such as *Ruminococcus torques group*, optimizes gut microbiota composition, and ultimately reduces the feed-to-gain ratio in broilers.

## Simple Summary

Reducing the feed-to-gain ratio remains a critical objective for cost reduction and efficiency enhancement in the livestock industry. This study investigated the effects of dietary supplementation with cyclic adenosine monophosphate nanoliposomes (Nano-cAMP) on growth performance, intestinal development, appetite-related hormone expression, and gut microbiota composition in broilers. Results demonstrated that Nano-cAMP supplementation significantly improved growth efficiency by reducing the feed-to-gain ratio. The treatment upregulated expression of cholecystokinin (CCK, an appetite-suppressing hormone) and secretin (a digestion-promoting hormone) genes, enhanced jejunal villus height, and increased the relative abundance of probiotic *Lactobacillus* species alongside cellulose-degrading bacterial populations in the intestinal microbiota. These findings indicate that Nano-cAMP supplementation effectively enhances growth performance and promotes intestinal health in broilers through multifaceted regulatory mechanisms.

## 1. Introduction

Cyclic adenosine monophosphate (cAMP), first discovered in 1958 by Earl W. Sutherland, plays a pivotal role in cellular signaling. Through molecular-level investigations into hormone action mechanisms, Sutherland established that hormones exert their regulatory effects on cells via cAMP, designating hormones as the “first messenger” and cAMP as the “second messenger” [1]. As a ubiquitous intracellular second messenger, cAMP is generally considered non-toxic and serves as a key mediator of signal transduction across diverse organisms. In microbial systems, cAMP participates in regulating gene expression associated with metabolic and growth processes. This signaling molecule not only modulates carbohydrate metabolism but also influences the integrity and function of microbial cell membranes, thereby affecting microorganism growth and reproduction [2]. Additionally, animal intestinal microbiota play crucial roles in food digestion and nutrient absorption. A balanced gut microbiome serves as the foundation for enhanced livestock productivity. Notably, cAMP demonstrates acid–base and thermal stability, with fermentation yields exceeding 7 g/L in current production systems [3]. However, the strong hydrophilicity of cAMP significantly limits its ability to traverse the lipid bilayer of cell membranes, resulting in poor bioavailability through oral administration. Early experimental evidence revealed that subcutaneous cAMP administration stimulates hypothalamic activity and enhances appetite in animal models [4]. Furthermore, parenteral cAMP administration has been shown to improve growth performance, increase lean muscle mass, enhance immune and digestive functions, and promote lactation in livestock [5]. Recent investigations have further established cAMP involvement in reproductive physiology [6], lipid metabolism regulation, and adipose tissue deposition [7], underscoring its multifaceted biological significance in animal systems. Despite these promising effects, practical applications in animal husbandry remain constrained by the impracticality of injection protocols and poor absorption via conventional feeding methods. To address these bioavailability challenges, researchers have explored the use of dibutyryl cyclic adenosine monophosphate (dbcAMP)-a lipophilic derivative with enhanced absorption properties-as a dietary supplement in swine nutrition. Experimental trials demonstrated that 20 mg/kg dietary supplementation effectively reduced adipose deposition in growing-finishing pigs [7]. Nevertheless, the predominant reliance on costly chemical synthesis methods for dbcAMP production continues to hinder its commercial-scale implementation.

Given liposomes’ well-documented advantages in targeted delivery efficiency and enhanced bioavailability [8,9], cyclic adenosine monophosphate (cAMP) encapsulated within nanoliposomes holds significant potential for improving growth performance and production efficiency in livestock. However, current research on cAMP nanoparticle-based formulations remains scarce. This study pioneers the dietary incorporation of Nano-cAMP liposomes to systematically evaluate their effects on broiler growth performance, intestinal morphology, and microbial composition. The findings aim to establish a scientific foundation for practical applications of nano-encapsulated cAMP in modern animal production systems.

## 2. Materials and Methods

All experimental procedures involving animals in this study were reviewed and approved by the Chongqing Academy of Animal Science Animal Ethics Committee (No. XKY-20240526, Chongqing, China).

### 2.1. Experimental Materials

Cyclic adenosine monophosphate (cAMP, analytical grade, ≥98% purity) was purchased from Shanghai Aladdin Biochemical Technology Co., Ltd., Shanghai, China, Nano-cAMP nanoliposomes were prepared using lecithin, cholesterol, citric acid monohydrate (C_6_H_8_O_7_·H_2_O), sodium citrate dihydrate (Na_3_C_6_H_5_O_7_·2H_2_O), chitosan (MW 200,000), and lactose. The encapsulation efficiency of cAMP in the nano-cAMP nanoliposomes was 84.73%. The preparation method of nano-cAMP nanoliposomes followed established protocols [10,11], with the detailed composition provided in Table 1.

### 2.2. Experiment Design

The experimental chickens were housed in cages measuring 54 cm × 50 cm × 50 cm (Long × Wide × High). The environment was maintained at 25 °C with 24 h continuous lighting. Vaccinations were administered following a standard immunization protocol, with food and water provided allodially throughout the trial period. Daily sanitation of the animal housing facility was conducted to minimize disease risks. Mortality and morbidity rates were recorded across different experimental groups during the rearing period.

A total of 108 21-day-old yellow-feathered female chicks were randomly assigned to three treatment groups (6 replicates per group, 6 hens per replicate) for a 21-day experimental period. The treatment groups consisted of: (1) Control group (CON): basal diet; (2) cAMP group: basal diet supplemented with 20 mg/kg cAMP; (3) Nano-cAMP group: basal diet supplemented with 0.37 g/kg Nano-cAMP liposomes (equivalent to 20 mg/kg cAMP). Diets were formulated according to NY/T 33-2004 [12] with calculated metabolizable energy values. Table 2 presents the detailed composition and nutritional profile of the experimental diets.

### 2.3. Sample Collection

On day 21 of the experiment, one chicken approximating the group mean weight was randomly selected from each enclosure and humanely euthanized via electrical stunning followed by exsanguination through carotid incision. Eighteen chicks were processed following this protocol. Post-euthanasia, the abdominal cavity was aseptically dissected to carefully excise jejunal mesenteric adipose tissue, with particular attention to preserving intestinal mucosal integrity. Identical 1 cm mid-jejunal segments were aseptically excised from standardized anatomical positions, rinsed with sterile physiological saline, and immersion-fixed in 4% paraformaldehyde for histomorphometric analysis. Luminal contents from mid-jejunal and mid-cecal regions were separately collected, flash-frozen in liquid nitrogen, and stored at −80 °C for subsequent microbial community analysis. Standardized 1 cm mid-duodenal sections were dissected, physiological saline-perfused to clear luminal contents, snap-frozen in liquid nitrogen, and maintained at −80 °C for qPCR quantification of gastrointestinal hormone mRNA. Residual gastrointestinal contents and adipose tissue were removed before systematically labeling proventriculus (glandular stomach), gizzard (muscular stomach), duodenum, jejunum, ileum, and cecum segments according to defined anatomical landmarks.

### 2.4. Detection Indicators and Methods

#### 2.4.1. Growth Performance

On days 1 and 21 of the experimental period, following a 12 h fasting period, all chicks were individually weighed by group. Daily feed consumption per cage was recorded throughout the trial. Subsequently, performance parameters including average daily gain (ADG), average daily feed intake (ADFI), and the feed-to-gain ratio were calculated. Average daily gain (g/d) = Total weight gain/(Number of chicks × Experimental days), Average daily feed intake (g/d) = Total feed intake/(Number of chicks per cage × Experimental days), Feed/gain ratio (g/g) = Total feed consumption/Total weight gain.

#### 2.4.2. Gastrointestinal Index

The relative weight indices of the gastrointestinal organs, including the glandular stomach, muscular stomach, duodenum, jejunum, ileum, and cecum, were determined. Digestive organ indices were calculated according to a previously described method [13], using the following formula: Organ index (%) = (organ weight/body weight before slaughter) × 100.

#### 2.4.3. Histological Morphology of Jejunum

The jejunum samples were fixed in 4% paraformaldehyde (Aladdin), sequentially dehydrated, cleared, and paraffin-embedded. Three tissue sections (5 μm) per sample were prepared (Leica microtome, RM2235, Wetzlar, Germany) and stained with hematoxylin (Sigma, Louis, MO, USA) and eosin (Sigma, Missouri, USA). Morphological examination (Leica optical microscope, DM500, 40×; Wetzlar, Germany) was performed equipped with a microscopic imaging system (Leica, DM1000, Wetzlar, Germany), with quantitative measurements of 10 villus heights and 10 crypt depths obtained from randomly selected fields per section (Image-Pro plus 6.0). Three sections were analyzed per sample. The villus height-to-crypt depth (V/C) ratio was subsequently calculated for statistical analysis. To minimize measurement bias, the procedure was performed by a researcher who was blinded to the group assignments.

#### 2.4.4. Expression of Duodenal Hormone mRNA

Total RNA was extracted from duodenal tissues using the Animal Tissue Total RNA Extraction Kit (TSP413, Qingke Biotechnology Co., Ltd., Beijing, China). cDNA was synthesized with the SynScript^®^ III RT SuperMix for qPCR (TSK314S, Qingke Biotechnology Co., Ltd.). Quantitative real-time PCR was performed using the ArtiCanCEO SYBR qPCR Mix (TSE401, Qingke Biotechnology Co., Ltd.) on a QuantStudio StepOne Plus Real-Time PCR System. The mRNA expression levels of cholecystokinin (CCK), ghrelin, secretin, and gastric inhibitory polypeptide (GIP) in the duodenum were analyzed according to the manufacturer’s recommended protocols. Glyceraldehyde-3-phosphate dehydrogenase (GAPDH) served as the reference gene, and relative gene expression was calculated using the 2^−△△Ct^ method. Primer sequences are listed in Table 3.

#### 2.4.5. Microbiota of Jejunum and Cecum

Jejunal and cecal contents were isolated, snap-frozen in liquid nitrogen, and stored at −80 °C. Total DNA from the samples was extracted with the TGuide S96 magnetic bead-based soil/fecal genomic DNA extraction kit (Tiangen Biotech Co., Ltd., Beijing, China). The 16S rRNA V3-V4 hypervariable regions were amplified using primers 338F (5′-ACTCCTACGGGAGGCAGCA-3′) and 806R (5′-GGACTACHVGGGTWTCTAAT-3′), followed by paired-end sequencing on an Illumina platform (Beijing Baimaike Cloud Technology Co., Ltd., Beijing, China). Operational taxonomic units (OTUs) were clustered at 97% nucleotide similarity using USEARCH (V10.0). Alpha diversity metrics and taxonomic composition (phylum/genus levels) were analyzed with QIIME 2. Microbial community differences were further evaluated via linear discriminant analysis effect size (LEfSe) on the Huttenhower Lab server (http://huttenhower.sph.harvard.edu/lefse/, accessed on 23 December 2024).

#### 2.4.6. Statistical Analysis of Data

Statistical analysis was performed using SAS 9.4 software. One-way analysis of variance (ANOVA) was conducted, followed by Duncan’s multiple range test for post hoc comparisons when significant differences were detected. The significance thresholds were defined as follows: *p* < 0.01 indicated statistically significant differences, while 0.01 ≤ *p* < 0.05 denoted marginally significant differences.

## 3. Results

### 3.1. Effects on Growth Performance

The effects of dietary interventions on broiler growth performance are summarized in Table 4. Compared with the CON group, the Nano-cAMP supplementation significantly reduced the feed-to-gain ratio (*p* < 0.05). No significant differences were observed in other growth parameters among the three experimental groups (*p* > 0.05).

### 3.2. Effects on Indices of Major Digestive Tract

The effects of dietary interventions on digestive organ indices are presented in Table 5. The cAMP group exhibited a significantly elevated duodenal index compared to both the Nano-cAMP and CON groups (*p* < 0.05). No statistically significant difference was detected between the Nano-cAMP and CON groups regarding duodenal index (*p* > 0.05). Furthermore, all other digestive organ parameters showed homogeneity across experimental groups (*p* > 0.05).

### 3.3. Effects on Jejunal Intestinal Tissue Morphology

**Figure 1:**
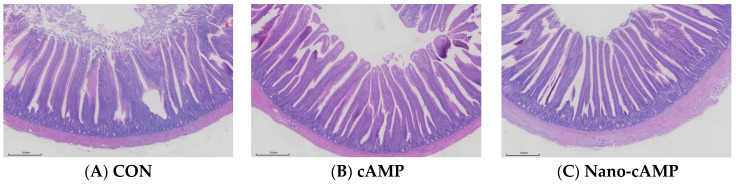
Representative histological micrographs of the jejunum. (**A**) CON: fed with basal diet; (**B**) cAMP: fed with basal diet + cAMP 0.02 g/kg; (**C**) Nano-cAMP: fed with basal diet + 0.37 g/kg Nano-cAMP liposomes. Bar = 500 μm.

The effects of dietary treatments on jejunal morphology are presented in Table 6. Villus height was significantly greater in the Nano-cAMP group compared with the CON group (*p* < 0.05). Consistent with the tabulated data, a marked increase in villus height was observed in the Nano-cAMP group (Figure 1). Although the cAMP group showed a 22.67% increase in villus height relative to the CON group, this difference did not reach statistical significance (*p* > 0.05). No significant difference in villus height was observed between the Nano-cAMP and cAMP groups (*p* > 0.05). Furthermore, dietary interventions had no significant effect on crypt depth or the villus height-to-crypt depth ratio in any experimental groups (*p* > 0.05).

### 3.4. Effects on Duodenal Intestinal Hormone mRNA Expression

As shown in Table 7, dietary treatments significantly influenced mRNA expression levels of CCK and secretin in the duodenum (*p* < 0.01). Compared with the CON group, both cAMP and Nano-cAMP groups demonstrated significantly elevated mRNA expression of CCK and secretin (*p* < 0.05). However, no significant difference was observed in CCK or secretin expression between the cAMP and Nano-cAMP groups (*p* > 0.05). Furthermore, mRNA levels of ghrelin and gastric inhibitory polypeptide (GIP) remained comparable across all experimental groups.

### 3.5. Effects on the Jejunal and Cecal Microbiota

**Figure 2:**
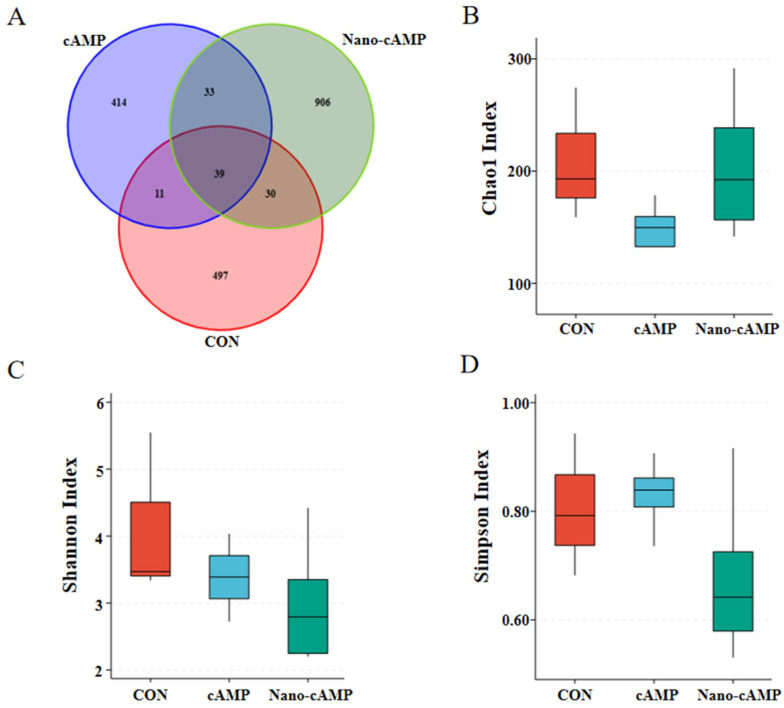
Effect of Nano-cAMP on the alpha diversity of jejunum microbiota. (**A**) Venn diagram of the OTUs distribution; (**B**) Chao1 Index of alpha diversity of jejunum microbiota; (**C**) Shannon Index; (**D**) Simpson Index. CON: fed with basal diet; cAMP: fed with basal diet + cAMP 0.02 g/kg; Nano-cAMP: fed with basal diet + 0.37 g/kg Nano-cAMP liposomes.

As revealed by the Venn diagram (Figure 2A), a total of 1930 operational taxonomic units (OTUs) were identified. The CON, cAMP, and Nano-cAMP groups exhibited 497, 414, and 906 unique OTUs, respectively, with 39 OTUs shared among all three groups. Dietary treatments did not significantly alter the alpha diversity indices (Chao1, Shannon, and Simpson) of the jejunal microbial communities in broilers (*p* > 0.05; Figure 2B–D).

**Figure 3:**
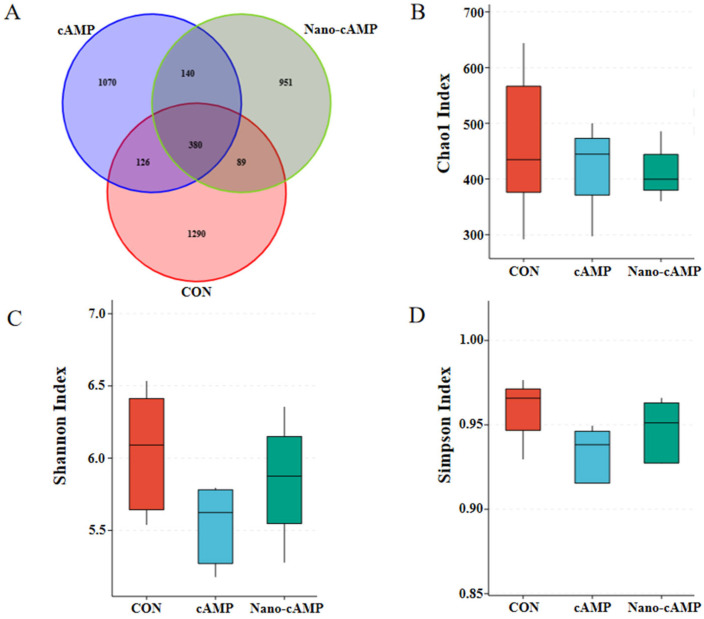
Effect of Nano-cAMP on the alpha diversity of cecum microbiota. (**A**) Venn diagram of the OTUs distribution; (**B**) Chao1 Index of alpha diversity of cecum microbiota; (**C**) Shannon Index; (**D**) Simpson Index. CON: fed with basal diet; cAMP: fed with basal diet + cAMP 0.02 g/kg; Nano-cAMP: fed with basal diet + 0.37 g/kg Nano-cAMP liposomes.

As shown in Figure 3A, a total of 4046 OTUs were analyzed by Venn diagram. The CON, cAMP, and Nano-cAMP groups contained 1290, 1070, and 951 unique OTUs, respectively, while 380 OTUs were shared among all three groups. The alpha diversity indices (Chao1, Shannon, and Simpson) of the cecal microbial community in broilers exhibited no significant differences among the dietary treatments (*p* > 0.05; Figure 3B–D).

As illustrated in Table 8, the three groups shared four dominant bacterial phyla at the phylum level—*Firmicutes*, *Proteobacteria*, *Bacteroidota*, and *Actinobacteriota*—collectively accounting for over 93% of total relative abundance. Compared to the CON group, both cAMP and Nano-cAMP groups exhibited significant increases in Firmicutes abundance (*p* < 0.05). *Proteobacteria* levels demonstrated differential reductions, with a more pronounced decrease in the cAMP group (*p* < 0.01) compared to the Nano-cAMP group (*p* < 0.05) relative to the CON group. Notably, significant declines (*p* < 0.05) were observed in *Gemmatimonadota* and *Chloroflexi* abundances across treatment groups. While Nano-cAMP administration enhanced *Actinobacteriota* representation, it concurrently reduced *Bacteroidota* levels along with six additional phyla. However, no significant compositional differences emerged between cAMP and Nano-cAMP groups at the phylum level.

As shown in Table 9, no significant differences in cecal microbiota composition were detected at the phylum level across groups (*p* > 0.05). *Firmicutes*, *Bacteroidota*, and *Actinobacteriota* constituted the predominant phyla in all groups, collectively accounting for over 98% of the total relative abundance. Compared to the CON group, both Nano-cAMP and cAMP groups displayed marked reductions in the relative abundance of *Firmicutes* and *Acidobacteriota*. A concurrent decrease in *Proteobacteria* abundance was also observed in two treatment groups. In contrast, *Bacteroidota* and *Actinobacteriota* exhibited increased relative abundances in the cAMP and Nano-cAMP groups relative to the control.

**Figure 4:**
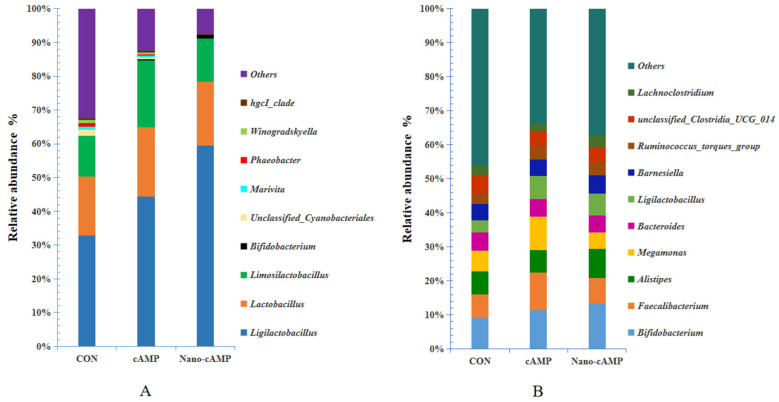
Composition of the microbiota at genus level: (**A**) jejunal microbiota; (**B**) cecal microbiota. CON: fed with basal diet; cAMP: fed with basal diet + cAMP 0.02 g/kg; Nano-cAMP: fed with basal diet + 0.37 g/kg Nano-cAMP liposomes.

As shown in Figure 4A, the combined relative abundance of *Ligilactobacillus*, *Lactobacillus*, *Limosilactobacillus*, and *Bifidobacterium* exceeded 60% at the genus level across all groups. Compared to the CON group, the cAMP and Nano-cAMP groups demonstrated a marked increase in the cumulative abundance of these four lactic acid-producing genera, with *Ligilactobacillus* and *Bifidobacterium* exhibiting particularly pronounced elevations. Conversely, taxa including *unclassified Cyanobacteriales*, *Marivita*, *Phaeobacter*, *Winogradskyella*, and the *hgcI_clade* (affiliated with the *Actinobacteria* phylum) were substantially reduced in both treatment groups relative to the control. Notably, these five genera showed near-complete depletion in the Nano-cAMP group. Figure 4B further revealed genus-level shifts: the cAMP and Nano-cAMP groups displayed modest increases in the relative abundance of *Bifidobacterium*, *Ligilactobacillus*, *Faecalibacterium*, and the *Ruminococcus_torques_group* compared to the CON group. In contrast, *unclassified_Clostridia_UCG_014* exhibited a marginal reduction in both treatment groups.

**Figure 5:**
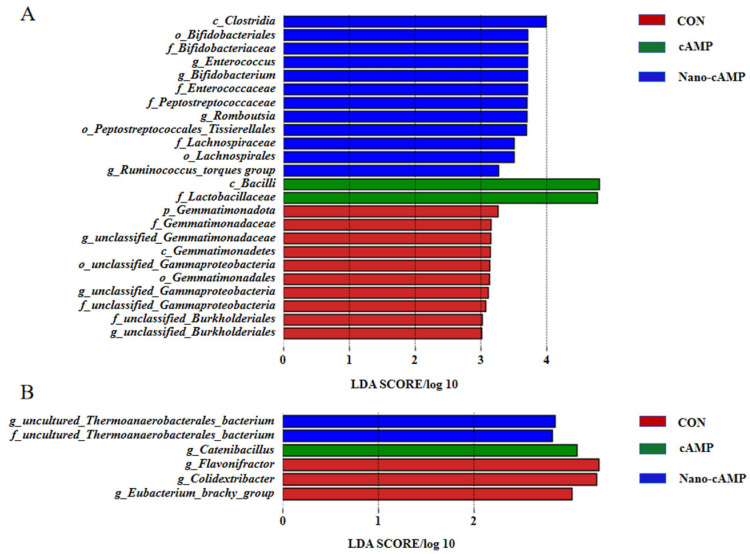
Histogram of linear discriminant analysis distribution of microbiota. (**A**) Jejunal microbiota; (**B**) Cecal microbiota. CON: fed with basal diet; cAMP: fed with basal diet + cAMP 0.02 g/kg; Nano-cAMP: fed with basal diet + 0.37 g/kg Nano-cAMP liposomes.

Figure 5A demonstrates the jejunal dominant genera identified by linear discriminant analysis. In the CON group, the predominant taxa were: *Gemmatimonadota* (*unclassified Gemmatimonadaceae* genus), *Proteobacteria* (*unclassified Gammaproteobacteria* genus), and *Proteobacteria* (*unclassified Burkholderiales* genus). The cAMP group was dominated by *Firmicutes* (*Bacilli* class; *Lactobacillaceae* family). The Nano-cAMP group exhibited multiple dominant genera: *Actinobacteriota* (*Bifidobacterium* genus), *Firmicutes* (*Enterococcus* genus), *Firmicutes* (Clostridia class; *Peptostreptococcales-Tissierellales* order; *Peptostreptococcaceae* family; *Romboutsia* genus), and *Firmicutes* (*Clostridia* class; *Lachnospirales* order; *Lachnospiraceae* family; *Ruminococcus_torques_group* genus). Figure 5B illustrates cecal microbial composition. The CON group showed dominance of *Firmicutes* (*Clostridia* class; *Peptostreptococcales-Tissierellales* order; *Anaerovoracaceae* family; *Eubacterium_brachy_group* genus), *Firmicutes* (*Colidextribacter* genus), and *Firmicutes* (*Flavonifractor* genus). *Catenibacillus* genus under the *Firmicutes* phylum predominated in the cAMP group. The Nano-cAMP group was characterized by *uncultured Thermoanaerobacterales bacterium* genus (*Firmicutes* phylum; *uncultured Thermoanaerobacterales* family).

Brief summary: Both cAMP and Nano-cAMP enhanced the relative abundance of probiotic genera, such as *Ligilactobacillus* and *Bifidobacterium*, as well as cellulose-degrading taxa *Faecalibacterium* and *Ruminococcus_torques_group*. Notably, Nano-cAMP additionally increased the relative abundance of an uncultured bacterium affiliated with the *Thermoanaerobacterales* family.

## 4. Discussion

### 4.1. Effects of Nano-cAMP Liposomes on Growth Performance of Broilers

Liposomes enhance absorption efficiency primarily through biomimetic delivery and transmembrane optimization mechanisms, and have demonstrated efficacy in improving vitamin and drug absorption [14,15]. Given the hydrophilic nature of cAMP, its limited ability to penetrate cell membranes [16] results in reduced bioavailability when administered orally. In a study by Li et al. [17], weaned piglets (average weight 3.31 kg) receiving 1.5 mg/day of calcium dibutyryl cyclic adenosine monophosphate (dbcAMP-Ca, a cAMP analog) for 10 days showed improved daily weight gain increased by 109.17% (*p* < 0.05). Interestingly, our findings revealed that neither directly adding cAMP or nano-cAMP liposomes significantly affected daily gain in broilers. However, the nano-cAMP formulation significantly reduced the feed-to-gain ratio. In healthy animals, improved feed efficiency reflects enhanced digestive capacity and intestinal microbial activity [18]. This suggests that dietary nano-cAMP liposomes may improve nutrient utilization, though the actual intestinal absorption of encapsulated cAMP requires further investigation.

Emerging evidence indicates that intracellular cAMP regulates growth-related gene expression via protein kinase A activation. Some researchers have demonstrated that dietary interventions combining prebiotics with cAMP elevation show potential for improving feed conversion efficiency. For instance, studies reported an 8.19–9.18% reduction in the feed/gain ratio of pigs (*p* < 0.05), a 9.22–9.68% increase in the relative breast muscle weight of broilers (*p* < 0.05), and a 3.01% increase in their relative thigh muscle weight (*p* < 0.01) [19,20,21]. Notably, Yue’s studies [22,23] on growing-finishing pigs demonstrated that elevated cAMP levels in high-efficiency groups downregulate lipogenesis genes while upregulating skeletal muscle development genes, confirming its regulatory role in growth processes. Although adenosine-based feed additives have shown growth-promoting effects [24], our study specifically highlights cAMP’s unique capacity to enhance growth performance. These findings collectively suggest that nano-cAMP formulations may offer novel strategies for improving dietary efficiency in poultry production.

### 4.2. Effects of Nano-cAMP Liposomes on Major Digestive Organ Indices and Jejunal Tissue Morphology

Digestion comprises two distinct processes: mechanical breakdown and enzymatic hydrolysis. As vital sites for nutrient processing and enzymatic absorption, digestive organ indices reflect developmental status, where higher indices correlate with enhanced digestive capacity [25]. This study demonstrated that dietary cAMP supplementation significantly elevated the duodenal index, while jejunal villus height showed no statistically significant increase. Notably, while nano-cAMP liposomes did not significantly alter duodenal index, they induced marked elevation in jejunal villus height. This differential effect may be attributed to the sustained-release properties and tissue-targeting capabilities inherent to liposomal formulations of cyclic adenosine monophosphate [26]. Both cAMP formulations promoted intestinal morphological development, thereby improving nutrient assimilation efficiency and consequently reducing the feed-to-gain ratio.

The jejunum, serving as the primary site of intestinal absorption, exhibits histological features representative of small intestinal development. Increased crypt depth typically indicates accelerated epithelial cell turnover, while elevated villus height and villus height/crypt depth ratio suggest enhanced mucosal differentiation with improved absorption capacity [27,28]. Although previous studies have established cAMP’s role in gastrointestinal smooth muscle relaxation [29], the current investigation provides novel evidence regarding exogenous cAMP’s impact on intestinal tissue morphogenesis, a previously unreported phenomenon in the literature.

### 4.3. Effects of Nano-cAMP Liposomes on Duodenal Enteric Hormone mRNA Expression

Cyclic adenosine monophosphate (cAMP), as an intracellular second messenger, mediates carbohydrate, protein, and lipid metabolism while coordinating hormone biosynthesis and physiological regulation. Our data revealed that dietary supplementation with both cAMP and nano-encapsulated cAMP significantly upregulated mRNA expression of CCK and secretin. Notably, ghrelin expression was elevated in both treatment groups, though without statistical significance. Previous studies indicate that CCK and ghrelin are key appetite-suppressing hormones in poultry, with elevated levels directly correlating with reduced feed intake [30,31]. This aligns with the observed downward trend in average daily feed consumption across treatment groups, albeit lacking statistical significance, thereby illustrating the multifactorial nature of appetite regulation involving digestive and growth-related pathways. Although a significant reduction in feed intake can negatively impact animal growth, it may improve the feed conversion ratio and benefit animal production. However, in this study, feed intake was not significantly reduced. Secretin enhances pancreatic juice secretion, and its upregulated expression in this study suggests improved digestive capacity in broilers. Mechanistically, cAMP-mediated regulation of enteric hormones involves two pathways: (1) activation of protein kinase A (PKA) through intracellular signaling cascades, modulating hormone CCK release [32]; (2) anatomical specificity, as CCK, secretin, and gastric inhibitory polypeptide are predominantly expressed in the duodenum [33], the primary site of enteric hormone production within the small intestine.

### 4.4. Effects of Nano-cAMP Liposomes on Intestinal Microbiota of Broilers

The gastrointestinal tract harbors a complex microbial ecosystem that critically regulates host physiology, including immunity, metabolism, and growth [34]. Gut microbial activities profoundly influence physiological homeostasis, exhibiting dual roles in health promotion and disease pathogenesis. Alpha diversity indices revealed microbial community characteristics: Chao1 estimates species richness (higher values = more taxa), whereas Shannon and Simpson indices reflect community diversity, influenced by both species richness and evenness [35,36].

Our findings indicate that neither conventional cAMP nor nano-cAMP significantly altered jejunal or cecal alpha diversity. However, both treatments enhanced the relative abundance of dominant taxa. Previous studies demonstrated that high-performing broilers exhibit jejunal microbiota dominated by *Firmicutes*, with elevated *Lactobacillus* and *Bifidobacterium* abundances and reduced pathogenic *Escherichia* populations [37]. Such probiotic dominance suppresses intestinal inflammation and enhances growth performance. Similarly, Qiu et al. [38] reported that ileal/cecal microbiota in healthy broilers showed increased *Lactobacillus* and *Bifidobacterium* with concurrent decreases in *Escherichia coli* and *Clostridium perfringens*, correlating with improved intestinal health and productivity.

Notably, both cAMP formulations increased jejunal *Firmicutes* abundance, particularly *Ligilactobacillus* and *Bifidobacterium*. Direct cAMP supplementation additionally elevated *Limosilactobacillus* abundance, while nano-cAMP specifically enhanced *Ruminococcus_torques_group* and *Romboutsia*. Furthermore, *Romboutsia* can metabolize complex carbohydrates into beneficial metabolites like short-chain fatty acids, oligosaccharides, and other prebiotic substances. [39]. Both treatments reduced jejunal *Gemmatimonadota* and *Proteobacteria* abundances. Although cecal *Firmicutes* slightly decreased, key beneficial genera (*Ligilactobacillus*, *Faecalibacterium*, *Ruminococcus_torques_group*, and *Bifidobacterium* [phylum *Actinobacteria*]) exhibited increased abundances. These microbiota shifts—probiotic enrichment coupled with pathogen suppression—positively correlate with enhanced immune competence and production efficiency [40,41,42], consistent with the observed reduction in feed-to-gain ratio.

The relative abundance of *unclassified_Thercoanaerobacterales_bacterium* in the cecum was significantly elevated by nano-cAMP liposome supplementation. Key microbial taxa including *Faecalibacterium* [43], *Ruminococcus_torques_group* [44], and *unclassified_Thercoanaerobacterales_bacterium* [45] function as primary degraders of lignocellulosic and other non-starch polysaccharides (NSPs). Their enzymatic hydrolysis releases metabolizable energy substrates for the host while concurrently reducing the feed-to-gain ratio. Notably, although direct cAMP administration markedly increased *Lactobacillaceae* (a beneficial family), it simultaneously elevated the pathogenic genus *Catenibacillus* (associated with gut diseases such as ulcer, constipation) [46]. Unclassified Bacterial taxa within *Gemmatimonadota* and *Proteobacteria* phyla, *Chloroflexi* (linked to white feces syndrome in shrimp) [47], *Eubacterium_brachy_group* (associated with human Glioblastoma) [48], *Colidextribacter* (Mice colitis) [49], and *Flavonifractor* (enriched in human irritable bowel syndrome) [50], are predominantly classified as opportunistic pathogens. These organisms may induce intestinal inflammation, impair nutrient absorption, and potentially increase the feed-to-gain ratio in control groups. Furthermore, the intestinal homeostatic equilibrium mediated by dominant probiotics (e.g., lactic acid bacteria) maintained clinical health status in experimental animals, with no overt disease manifestations observed during the trial period.

## 5. Conclusions

In conclusion, dietary supplementation with 0.37 g/kg nano-cAMP liposomes enhances broiler performance through multifaceted mechanisms: (1) upregulating duodenal CCK and secretin gene expression; (2) increasing jejunal villus height to amplify nutrient absorption capacity; (3) enriching beneficial microbiota and cellulolytic taxa, thereby optimizing intestinal ecosystem functionality. These synergistic effects collectively improve absorption efficiency and reduce the feed-to-gain ratio.

## Figures and Tables

**Table 1:** Raw material composition and content of Nano-cAMP.

Ingredients (%)	Content
cAMP	5.41
Lecithin	22.70
Cholesterol	4.32
Citric acid monohydrate(C_6_H_8_O_7_·H_2_O)	22.16
Sodium citrate dihydrate (Na_3_C_6_H_5_O_7_·2H_2_O)	7.57
Chitosan(MW 200,000)	10.81
Lactose	27.03
Total	100.00

**Table 2:** Composition and nutrient levels of basal diets (feed basis).

Items	Content/%
Ingredients	
Corn	37.50
Wheat	25.00
Soybean meal	2.50
Wheat bran	15.00
Corn protein meal	12.50
Soybean oil	2.00
Limestone	1.25
CaHPO_4_	2.15
*L*-Lysine·H_2_SO_4_	0.84
*DL*-Methionine	0.11
NaCl	0.25
Choline chloride	0.10
NaHCO_3_	0.10
Phytase	0.02
Cellulase	0.05
Premix ^1^	0.63
Total	100.00
Nutrient levels	
Metabolizable energy/(MJ/kg)	12.12
Crude protein	18.70
Lysine	0.91
Methionine	0.45
Methionine + Cysteine	0.70
Calcium	1.10
Total phosphorus	0.72

^1^ Premix provided the following per kilogram of diets: Vitamin A 12,000 IU, vitamin D3 3000 IU, vitamin E 30 IU, vitamin K3 4.8 mg, vitamin B1 3 mg, vitamin B2 9.6 mg, vitamin B6 6 mg, vitamin B12 0.03 mg, niacin 60 mg, *D*-calcium pantothenate 18 mg, folic acid 1.5 mg, *D*-biotin 0.17 mg, Fe 60 mg, Cu 6 mg, Mn 80 mg, I 0.70 mg, Se 0.30 mg.

**Table 3:** Primer sequences.

Gene Name ^1^	Primer Sequence (5′-3′)	Product Size (bp)
CCK	F: ACTGGGAGGTTCTCTGTCCT	155
	R: CTGTTGCTATCGCCTGCTGT	
Ghrelin	F: AACAGCAAGATTACATCGCCG	169
	R: CATCTTCTCCAGTGCTTGTCC	
Secretin	F: AGCAATCCAAGCCTGGTCA	132
	R: AGCTTCCTTGGCATCGTTGT	
GIP	F: ATGCACAGACGCTACTCGG	153
	R: AGGCTCGGCTTCTCTCTTGT	
GAPDH	F: ATGGGCACGCCATCACTATC	138
	R: CACCACCCTTCAGATGAGCC	

^1.^ CCK: cholecystokinin; GIP: gastric inhibitory polypeptide; GAPDH: Glyceraldehyde-3-phosphate Dehydrogenase.

**Table 4:** Effect of Nano-cAMP on growth performance of broilers.

Items ^1^	CON	cAMP	Nano-cAMP	SEM	*p*-Value
Initial body weight/g	354.00	353.75	352.92	3.042	0.989
Final body weight/g	1096.00	1083.67	1128.75	10.390	0.138
Feed/gain ratio	2.39 ^a^	2.33 ^ab^	2.27 ^b^	0.019	0.025
Average daily feed intake/g	84.43	80.08	83.93	1.129	0.253
Average daily gain/g	35.33	34.42	36.94	0.494	0.122

^1^ CON: fed with basal diet; cAMP: fed with basal diet + cAMP 0.02 g/kg; Nano-cAMP: fed with basal diet + 0.37 g/kg Nano-cAMP liposomes. In the same row, values with no letter or the same letter superscripts mean no significant difference (*p* > 0.05), while values with different small-letter superscripts mean a significant difference (*p* < 0.05).

**Table 5:** Effects of Nano-cAMP on indices of major digestive tract of broilers.

Items ^1^	CON	cAMP	Nano-cAMP	SEM	*p*-Value
Glandular gastric index	0.40	0.41	0.43	0.022	0.907
Muscle stomach index	1.33	1.44	1.34	0.032	0.301
Duodenal index	0.46 ^b^	0.61 ^a^	0.49 ^b^	0.026	0.011
Jejunal index	0.91	1.10	0.99	0.044	0.264
Ileal index	0.76	0.70	0.70	0.026	0.631
Cecal index	0.34	0.33	0.31	0.013	0.741

^1^ CON: fed with basal diet; cAMP: fed with basal diet + cAMP 0.02 g/kg; Nano-cAMP: fed with basal diet + 0.37 g/kg Nano-cAMP liposomes. In the same row, values with no letter or the same letter superscripts mean no significant difference (*p* > 0.05), while values with different small-letter superscripts mean a significant difference (*p* < 0.05).

**Table 6:** Effects of Nano-cAMP on intestinal tissue morphology of the jejunum.

Items ^1^	CON	cAMP	Nano-cAMP	SEM	*p*-Value
Villous height/μm	1084.57 ^b^	1330.44 ^ab^	1423.81 ^a^	56.947	0.033
Crypt depth/μm	155.59	181.06	183.75	10.752	0.570
Villus height/Crypt depth	7.11	7.52	7.91	0.331	0.660

^1^ CON: fed with basal diet; cAMP: fed with basal diet + cAMP 0.02 g/kg; Nano-cAMP: fed with basal diet + 0.37 g/kg Nano-cAMP liposomes. In the same row, values with no letter or the same letter superscripts mean no significant difference (*p* > 0.05), while values with different small-letter superscripts mean a significant difference (*p* < 0.05).

**Table 7:** Effect of Nano-cAMP on mRNA expression of duodenal intestinal hormones.

Items ^1^	CON	cAMP	Nano-cAMP	SEM	*p*-Value
CCK	1.00 ^b^	5.24 ^a^	4.81 ^a^	0.695	0.003
Ghrelin	1.01	1.03	1.31	0.078	0.209
Secretin	1.02 ^b^	2.65 ^a^	2.31 ^a^	0.320	0.006
GIP	1.01	0.90	1.10	0.053	0.400

^1^ CON: fed with basal diet; cAMP: fed with basal diet + cAMP 0.02 g/kg; Nano-cAMP: fed with basal diet + 0.37 g/kg Nano-cAMP liposomes. In the same row, values with no letter or the same letter superscripts mean no significant difference (*p* > 0.05), while values with different small-letter superscripts mean a significant difference (*p* < 0.05). CCK: cholecystokinin; GIP: gastric inhibitory polypeptide.

**Table 8:** Composition of the jejunum microbiota at the phylum level.

Items ^1^	CON	cAMP	Nano-cAMP	SEM	*p*-Value
*Firmicutes*	83.640 ^b^	97.526 ^a^	95.152 ^a^	2.110	0.022
*Proteobacteria*	5.350 ^a^	1.013 ^b^	1.434 ^b^	0.629	0.007
*Bacteroidota*	2.729	0.310	0.468	0.435	0.063
*Actinobacteriota*	1.320	0.535	2.138	0.321	0.085
*Cyanobacteria*	0.810	0.022	0.052	0.154	0.097
*Acidobacteriota*	1.133	0.097	0.126	0.188	0.058
*Chloroflexi*	0.483 ^a^	0.068 ^b^	0.084 ^b^	0.069	0.029
*Myxococcota*	0.779	0.037	0.040	0.160	0.150
*Gemmatimonadota*	0.354 ^a^	0.010 ^b^	0.055 ^b^	0.050	0.010
*Others*	3.403 ^a^	0.382 ^b^	0.450 ^b^	0.474	0.012

^1^ CON: fed with basal diet; cAMP: fed with basal diet + cAMP 0.02 g/kg; Nano-cAMP: fed with basal diet + 0.37 g/kg Nano-cAMP liposomes. In the same row, values with no letter or the same letter superscripts mean no significant difference (*p* > 0.05), while values with different small-letter superscripts mean a significant difference (*p* < 0.05).

**Table 9:** Composition of the cecum microbiota at the phylum level.

Items ^1^	CON	cAMP	Nano-cAMP	SEM	*p*-Value
*Firmicutes*	70.692	68.167	65.144	2.133	0.597
*Bacteroidota*	18.877	18.953	20.350	1.806	0.940
*Actinobacteriota*	9.362	11.655	13.544	1.406	0.506
*Desulfobacterota*	0.535	0.540	0.666	0.104	0.861
*Proteobacteria*	0.296	0.154	0.161	0.054	0.511
*Campylobacterota*	0.078	0.414	0.045	0.087	0.163
*Cyanobacteria*	0.058	0.064	0.071	0.029	0.985
*Verrucomicrobiota*	0.009	0.011	0.009	0.003	0.941
*Acidobacteriota*	0.016	0.005	0.002	0.003	0.078
*Others*	0.078	0.036	0.008	0.014	0.131

^1^ CON: fed with basal diet; cAMP: fed with basal diet + cAMP 0.02 g/kg; Nano-cAMP: fed with basal diet + 0.37 g/kg Nano-cAMP liposomes. In the same row, values with no letter or the same letter superscripts mean no significant difference (*p* > 0.05), while values with different small-letter superscripts mean a significant difference (*p* < 0.05).

## Data Availability

The data presented in this study are available in the article.
